# Modified acupuncture therapy, long-term acupoint stimulation versus sham control for weight control: a multicenter, randomized controlled trial

**DOI:** 10.3389/fendo.2022.952373

**Published:** 2022-07-28

**Authors:** Liang Dai, Miao Wang, Ke-Pei Zhang, Lin Wang, Hui-Min Zheng, Chun-Bo Li, Wen-Jun Zhou, Shi-Gao Zhou, Guang Ji

**Affiliations:** ^1^ Institute of Digestive Diseases, Longhua Hospital, Shanghai University of Traditional Chinese Medicine, Shanghai, China; ^2^ Clinical Research Academy, Peking University Shenzhen Hospital, Peking University, Shenzhen, China; ^3^ Department of Internal Medicine of Traditional Chinese Medicine, Longhua Hospital, Shanghai University of Traditional Chinese Medicine, Shanghai, China; ^4^ Department of Internal Medicine of Traditional Chinese Medicine, Shanghai Pudong New Area Peoples’ Hospital, Shanghai, China; ^5^ Department of Acupuncture and Moxibustion, Shanghai Municipal Hospital of Traditional Chinese Medicine, Shanghai University of Traditional Chinese Medicine, Shanghai, China; ^6^ Shanghai Key Laboratory of Psychotic Disorders, Shanghai Mental Health Center, Shanghai Jiao Tong University School of Medicine, Shanghai, China

**Keywords:** acupuncture, long-term acupoint stimulation, weight control, visceral adipose tissue, subcutaneous adipose tissue, randomized controlled trial

## Abstract

**Objective:**

Long-term acupoint stimulation (LAS), also called embedding acupuncture, is a modified acupuncture technique. The preliminary results have demonstrated its efficacy in body-weight control. However, the low quality of available trials limited its application. This study aimed to evaluate the efficacy and safety of LAS in body-weight control by using a randomized, parallel, sham-controlled clinical trial design.

**Methods:**

This was a randomized, single-blind, sham-controlled clinical trial including 84 adult participants (18–60 years) with a body mass index (BMI) of ≥ 24 kg/m^2^ conducted in three general hospitals in Shanghai, China. Participants were equally assigned to receive LAS or sham LAS (SLAS) once per 10 days, eight times in total. After completion, an additional intervention with a 3-month follow-up period was set to examine the continued effect of LAS. The primary outcome was the change in body weight from baseline to treatment endpoint within the intention-to-treat (ITT) analysis. Secondary outcomes contained changes in waist-to-hip ratio (WHR), lipid metabolism, and visceral and subcutaneous adipose tissues.

**Results:**

From 14 May 2018 to 03 November 2019, 84 participants out of 201 screened individuals met the eligibility criteria, were randomized, and were analyzed (42 participants in each group). From baseline to treatment endpoint, the body-weight reduction in the LAS group was significantly larger than in the sham control (net difference: 1.57 kg, 95% CI: 0.29–2.86, *p* = 0.012). The superior weight reduction effect persisted in the follow-up period (net difference: 3.20 kg, 95% CI: 1.17–5.21, *p* = 0.001). LAS therapy also showed improvement in triglyceride and subcutaneous adipose tissue (SAT) compared with sham control. One participant in the LAS group reported a slightly uncomfortable and tingling sensation after the additional intervention. No other adverse events (AEs) were documented.

**Conclusion:**

LAS, a modified acupuncture technique, is safe and effective in body-weight control. It could be used as an alternative choice to classical acupuncture for obesity management.

**Clinical Trial Registration:**

[www.chictr.org.cn], identifier [ChiCTR1800015498].

## Introduction

Due to an unhealthy lifestyle, overweight and obesity have affected more than one-third of the Chinese population (1). Along with high prevalence, the level of body mass index (BMI) has also presented a consistently increasing trend in recent decades (2). Abundant studies have demonstrated that obesity is an independent risk factor for nonalcoholic fatty liver disease, cardiovascular diseases, and even certain types of cancers (3–5). Hence, active management of overweight and obesity is crucial in daily practice.

Conventional strategies for body-weight control include lifestyle modification, use of chemical agents, and surgical interventions (6–8). Certain pharmacotherapies, in addition to lifestyle modification, indeed present a significant effect on weight loss (9, 10). However, majority of people would experience various adverse events (AEs) as well as benefits in health outcomes except for the quality of life, which was still lacking (11). Surgical treatment is not a conventional option and could only be considered for patients with a BMI of ≥35 kg/m^2^ (12). Hence, patients and clinicians would introduce alternative therapies to body-weight management, and acupuncture is one of the representative options.

Various studies have illustrated that acupuncture can significantly reduce body weight and BMI compared with sham control (13, 14). However, based on the synthesized data, the effect size of classical acupuncture was not enough to achieve the goal of weight loss (7). Thus, different techniques have been utilized to modify conventional acupuncture. Long-term acupoint stimulation (LAS), also called embedding acupuncture, is a kind of modified technique developed based on extended needle retention. By implanting compatible and self-degradable materials into acupoints, the acupuncture sensation would be amplified and prolonged, and therefore, the efficacy would be enhanced theoretically (15). Previous systematic reviews have summarized the current evidence of LAS in controlling body weight (16, 17). However, the conclusion should be interpreted with caution due to the low quality of included trials and high heterogeneity.

In order to verify the effectiveness and safety of LAS for weight control, we conducted this rigorous multicenter, randomized clinical trial using sham control as the comparator. The results of this trial would also serve as foundational data for further potential head-to-head trials versus conventional acupuncture.

## Materials and methods

### Study design

This was a 6-month, multicenter, single-blind, sham-controlled, parallel clinical trial conducted in Longhua Hospital Affiliated to Shanghai University of Traditional Chinese Medicine, People’s Hospital of Pudong District of Shanghai and Shanghai Municipal Hospital of Traditional Chinese Medicine. Qualified participants were randomly assigned to LAS or sham LAS (SLAS) arms according to a ratio of 1:1. LAS or SLAS was given once per 10 days, eight times in total. After completion of treatment, an additional intervention with a 3-month follow-up period was set to assess the continued effect of LAS.

The study protocol was approved by the Clinical Trial Institutional Review Board (IRB) of Longhua Hospital Affiliated to Shanghai University of Traditional Chinese Medicine (No. 2016LCSY321) and registered at the Chinese Clinical Trial Registry (www.chictr.org.cn, No. ChiCTR1800015498) on 10 April 2018. The execution of the trial strictly followed the Helsinki Declaration and Good Clinical Practice Guideline. The reporting of this clinical trial followed the requirements of the Consolidated Standards of Reporting Trials (CONSORT) and Standards for Reporting Interventions in Controlled Trials of Acupuncture (STRICTA) statements (18, 19), and the corresponding checklists can be found in **Supplementary Tables S1, S2**.

### Participants

All participants were recruited publicly through advertisements on social media. Potential subjects could choose one of the three study centers to participate in the trial. The definition of overweight and obesity was based on the Guidelines for Prevention and Control of Overweight and Obesity in Chinese Adults, specifically a BMI of ≥24 kg/m^2^ (20). Other inclusion criteria were age, from 18 to 60 years old, and voluntary informed consent. The exclusion criteria included pregnancy and lactation; allergy to embedding material, namely, poly-*p*-dioxanone (PPDO); severe comorbidities such as cardiovascular, hepatic, renal, or other primary diseases; and mental illness. After eligibility, informed consent was obtained from the participants and then confirmed.

### Interventions

According to previous systematic reviews and consultations with specialists (13, 16), the following acupoints were chosen: Zhongwan (CV12), Tianshu (ST25), Fenglong (ST40), and Pishu (BL20). Previous reports have demonstrated their functions in regulating appetite and energy metabolism (21–23). According to traditional Chinese medicine (TCM) theory, these acupoints have the functions of regulating the qi activity and invigorating the spleen to eliminate dampness (24). The specific anatomical locations are described in **Table 1** and shown in **Supplementary Figure S1**.

**Table 1:** Locations of selected acupoints.

Acupoints	Anatomical location
Zhongwan (CV12)	On the anterior median line of the upper abdomen, 4.0 cun above the umbilicus
Tianshu (ST25)	On the middle of the abdomen, 2 cun lateral to the umbilicus
Fenglong (ST40)	On the anterior aspect of the lower leg, 8 cun superior to the external malleolus, lateral to ST38, two finger-breadth (middle finger) from the anterior crest of the tibia
Pishu (BL20)	On the back, 1.5 cun lateral to the lower border of the spinous process of the 11th thoracic vertebra

The bioabsorbable PPDO suture was chosen as the embedding material. PPDO has been widely used in various surgical procedures (25). It could be completely degraded into water and carbon dioxide in approximately half a year in the body (26, 27). Previous studies have illustrated its satisfactory biocompatibility (28, 29); hence, a low level of inflammatory response was anticipated during the LAS treatment.

The LAS procedure was completed by professional acupuncturists with at least 10 years of clinical practice experience. The embedding needle (size no. 7) was composed of needle tubing and stylet. Before inserting the stylet, a microbic PPDO suture (size: 3–0) was first placed in the tip of a disposable embedding needle. The reason for choosing this size was based on researchers’ clinical experience, specifically that previous patients who received this size of suture reported better treatment response and less discomfort sensation. Next, the needle was inserted at a depth of 1.5–2.0 cm into the acupoints. The depth was consistent with routine acupuncture insertion depth in previous trials (30). Choosing this depth matched the aim of LAS therapy, namely, prolonging the needle sensation and avoiding the introduction of other biases. The position of the needle tip should be between the skin and muscle. After obtaining needle sensation (feelings of soreness, numbness, fullness, and heaviness in the acupoints, also defined as “de-qi”), the acupuncturist would gradually pull out the tubing and push in the stylet at the same time. This simultaneous operation would ensure the PPDO suture underneath the acupoint. Afterward, the embedding needle was withdrawn, the pinhole was pressed gently with sterile cotton and then taped. The size of the implanted PPDO suture and the key procedures of LAS therapy could be found in **Supplementary Figures S2, S3**. For the SLAS arm, the treatment procedure was the same, except that no PPDO suture was in the embedding needle. In order to guarantee the blinding of participants, they were required to keep the supine position first to finish the procedure in CV12, ST25, and ST40. They were asked to keep looking ahead during the procedure and to look down at their body was strictly forbidden. They were then asked to turn over to the prone position to finish the procedure in BL20, which was exactly the vision-blind area of the participants. All participants were informed to gently massage the acupoints before and after meals for 2 min.

The procedure was carried out once every 10 days, eight times in total. The treatment interval could be adjusted by the acupuncturist properly when encountering exceptional circumstances such as a menstrual period. The treatment scheme was established based on a previous systematic review and specialist consultations (31). To be specific, we asked about the patients’ feelings of sensation during LAS therapy in our daily practice. Most of them reported that the sensation disappeared in approximately 1–2 weeks. Based on the preliminary data, we determined the treatment interval as 10 days after consultation with specialists. For the treatment duration, we noticed that a 12-week period was chosen in the majority of clinical trials regarding body-weight control. According to the previous treatment interval, we ultimately set the treatment strategy as once every 10 days, eight times in total. After completion of treatment, an additional intervention with a 3-month follow-up period was set to examine the continued effect.

Lifestyle modification plays an important role in body-weight management. In order to evaluate the effect of LAS in a real-world setting, participants were informed to follow the lifestyle modification requirements. Dramatic changes in calorie intake and/or physical activities were not allowed. Healthy lifestyle suggestions were provided in every follow-up visit according to overweight and obesity management guidelines, including limiting calorie intake and increasing physical exercise (20). To be specific, the total calorie of a daily diet should be around 1,660 kcal. Moderate physical exercise should be performed four times per week at least, and the whole time should be above 150 min. The suggestions were communicated through brochures and oral education. During every visit, the clinical investigator would ask participants about the execution of lifestyle modification. Refusal to follow lifestyle suggestions was considered a protocol violation.

### Outcomes

Participants were required to complete body measurements and scheduled laboratory tests during every visit before treatment or consultation. The primary outcome was the change in body weight after treatment. Secondary outcomes included the change in waist-to-hip ratio (WHR), total cholesterol (TC), triglyceride (TG), high-density lipoprotein cholesterol (HDL-C), and low-density lipoprotein cholesterol (LDL-C). In addition, on the principle of voluntariness, we recruited some participants to assess the change in body adipose tissue by using magnetic resonance imaging. The specific assessment method is documented in **Supplementary File 1**. The exploratory outcomes were the changes in visceral adipose tissue (VAT) and subcutaneous adipose tissue (SAT).

Safety assessments involved blood routine tests, urine routine tests, stool routine tests, hepatic and renal function tests, and 12-lead electrocardiograms. AEs were documented during the whole study. Necessary medical interventions were also given to patients experiencing AEs.

### Randomization and blinding

Block randomization was conducted in the ratio of 1:1 according to the random number table generated from NCSS software 11 (NCSS, LLC, Kaysville, UT, USA). An independent research assistant was responsible for the randomization and preparation of treatment assignment envelopes. The opaque sealed envelopes only had coherent randomization numbers on the surface and could not be opened until the eligibility of participants was confirmed. Clinical practitioners were responsible for informed consent and participant screening. All potential participants were informed about the rationale of LAS therapy, the detailed therapy process, and that they would be randomly allocated to a real LAS group or sham control. The allocation was not revealed until the study’s completion. For participants who received sham LAS therapy, they can obtain a compensatory nine-time LAS therapy according to their willingness. Eligible participants were to confirm the above information before enrolment. After eligibility confirmation of the participants, the clinical practitioner were to open the envelope based on the inclusion order and perform the procedure accordingly. Participants were blinded during the whole study. Apart from position requirements during treatment, the implanting material made the blinding more reliable. To be specific, before the execution of this trial, we did a survey of patients who had been treated with LAS therapy. The survey indicated that the size of the PPDO suture (size 3–0) we selected was well-tolerated, and most of the patients reported no obvious sense of the implanting material. Due to the specificity of acupuncture trials, the practitioners could not be blinded. Other investigators, including coordinators, assessors, statisticians, and participants were blinded to the specific assignments.

### Statistical analysis

The sample size calculation was based on the primary outcome. According to our pilot study, the mean reduction of body weight by SAT was 6.61 kg with a standard deviation (SD) of 10.53 (unpublished data). NCSS software (NCSS, LLC, Kaysville, UT, USA) was used to estimate the sample size. In order to yield an 80%-statistical power with a two-sided significance level of 0.05, 38 participants per group were needed. Furthermore, assuming a dropout rate of 10%, a total of 84 participants were determined.

All outcome measures were evaluated based on the intention-to-treat (ITT) principle. The analysis set was composed of all randomized participants. The missing data were filled out using the last-observation-carried-forward method. Furthermore, for the follow-up data, the analysis set was defined as the participants who underwent evaluation at both the baseline and corresponding follow-up timepoints. The R software (Version 3.6) was utilized to conduct statistical analysis. Data were presented in mean values with an SD or as a number with a percentage. The primary outcome, change in body weight, was evaluated by repeated-measures analysis of variance. Baseline differences between groups and other outcome variables were assessed by Student’s *t*-test, Chi-square test, or Mann–Whitney *U* test according to the data characteristics. Significance was defined as a two-tailed value of 0.05.

## Results

### Baseline characteristics

**Figure 1:**
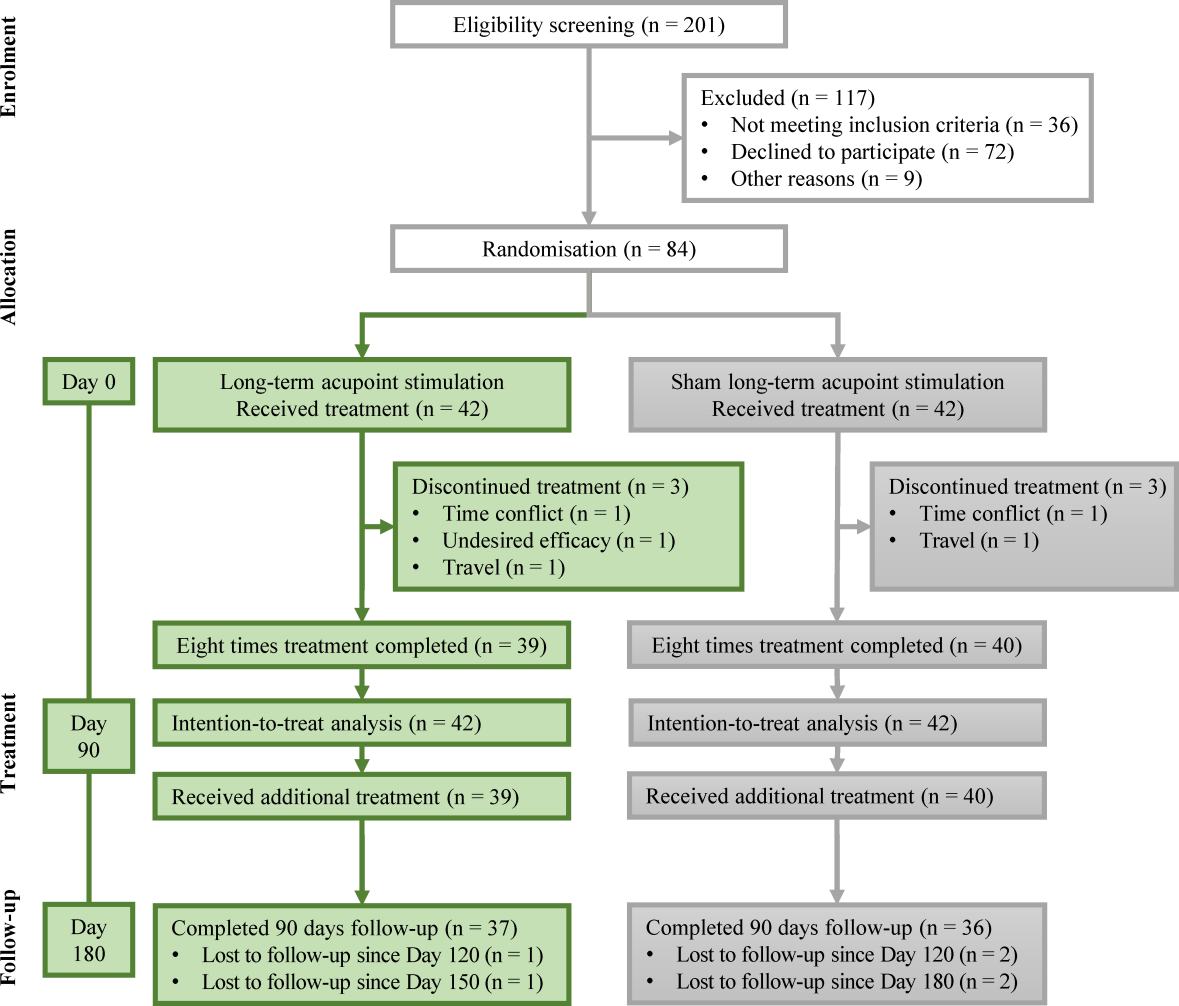
Participants’ flowchart.

From 14 May 2018 to 03 November 2019, 201 individuals were screened. Among them, 84 participants met the eligibility requirements and were randomized. Finally, 79 participants completed the study. The reasons for withdrawal included undesired efficacy (*n* = 1), time conflict (*n* = 2), and travel (*n* = 2). Treatment adherence for the whole intervention was 94%. The participant flowchart is shown in **Figure 1**. The baseline characteristics of the included participants are presented in **Table 2**. No statistically significant difference was found between the two groups.

**Table 2:** Baseline characteristics of the intention-to-treat (ITT) population.

	LAS (*n* = 42)	SLAS (*n* = 42)	*p*-value
Gender			
Male	14 (33.33%)	19 (45.24%)	0.26
Female	28 (66.67%)	23 (54.76%)	
Age (years)	33.05 (6.60)	36.17 (9.71)	0.25
Body weight (kg)	83.99 (20.33)	85.20 (13.46)	0.69
BMI (kg/m^2^)	29.98 (4.78)	30.18 (3.70)	0.38
Waist circumference (cm)	99.79 (13.62)	101.21 (10.19)	0.58
Hip circumference (cm)	107.69 (10.07)	108.31 (7.42)	0.75
WHR	0.92 (0.07)	0.93 (0.07)	0.50
TC (mmol/L)	4.64 (0.91)	4.76 (0.99)	0.99
TG (mmol/L)	1.80 (1.52)	1.38 (0.55)	0.06
LDL-C (mmol/L)	3.11 (0.68)	3.24 (0.77)	0.51
HDL-C (mmol/L)	1.21 (0.24)	1.29 (0.27)	0.31
Comorbidities			
Hypertension	6 (14.29%)	4 (9.52%)	0.50
CHD	0 (0%)	0 (0%)	1.00
Dyslipidemia	2 (4.76%)	2 (4.76%)	1.00
NAFLD	9 (21.43%)	8 (19.05%)	0.79
CKD	0 (0%)	0 (0%)	1.00

Data are in n (%) or mean (SD) values.

BMI, body mass index; CHD, coronary heart disease; CKD, chronic kidney disease; HDL-C, low-density lipoprotein cholesterol; LAS, long-term acupoint stimulation; LDL-C, low-density lipoprotein cholesterol; NAFLD, nonalcoholic fatty liver disease; SLAS, sham long-term acupoint stimulation; TC, total cholesterol; TG, triglyceride; WHR, waist-to-hip ratio.

### Changes in body weight and BMI

**Figure 2:**
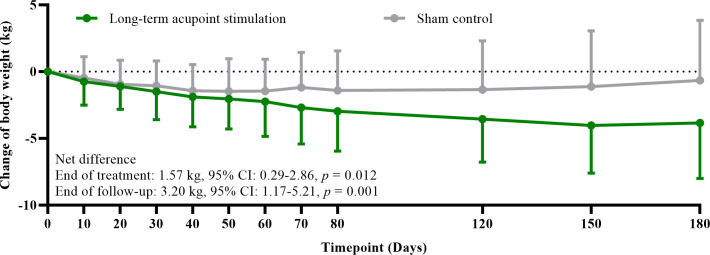
Change of body weight from baseline to 180 days follow-up.

The mean reduction of body weight was 2.97 kg (SD: 2.98) after eight times of treatment in the LAS group, while it was 1.40 kg (SD: 2.95) in the sham control; a higher amplitude was noticed in the LAS group (net difference: 1.57 kg, 95% confidence interval (CI): 0.29–2.86, *p* = 0.012) (**Table 3**). The weight reduction effect persisted in the follow-up period. The mean reduction of body weight was 3.84 kg (SD: 4.17) from baseline to follow-up endpoint in the LAS group, while it was 0.65 kg (SD: 4.49) in the sham control; a larger decrease was still found in the LAS group (net difference: 3.20 kg, 95% CI: 1.17–5.21, *p* = 0.001) (**Table 2**). The change in body weight along with time in the two groups is shown in **Figure 2**. At the end of treatment, 12 (28.6%) and 6 (14.3%) participants achieved at least a 5% reduction in body weight in the LAS group and sham control, respectively.

**Table 3:** Change of body weight and body mass index during the whole trial.

	Baseline to treatment endpoint			
	LAS (*n* = 42)	SLAS (*n* = 42)	Net difference and 95% CI	*p*-value
Body weight (kg)	2.97 (2.98)	1.40 (2.95)	1.57 (0.29, 2.86)	0.012
BMI (kg/m^2^)	1.07 (1.10)	0.49 (1.04)	0.58 (0.12, 1.05)	0.010
	**Baseline to follow-up endpoint**			
	**LAS (*n* = 37)**	**SLAS (*n* = 36)**	**Net difference and 95% CI**	***p*-value**
Body weight (kg)	3.84 (4.17)	0.65 (4.49)	3.20 (1.17, 5.21)	0.001
BMI (kg/m^2^)	1.39 (1.56)	0.22 (1.59)	1.16 (0.42, 1.89)	0.001

Data are in mean (SD) values.

BMI, body mass index; LAS, long-term acupoint stimulation; SLAS, sham long-term acupoint stimulation.

Similarly, the mean change in BMI was −1.07 kg/m^2^ (SD: 1.10) in the LAS group after treatment. The reduction was significantly higher than the SLAS group (−0.49 kg/m^2^ (SD: 1.04)) (net difference: 0.58 kg/m^2^, 95% CI: 0.12–1.05, *p* = 0.010) (**Table 2**). From beginning to follow-up endpoint, the reduction of BMI was still 1.39 kg/m^2^ (SD: 1.56) in the LAS group, while it was 0.22 kg/m^2^ (SD: 1.59) in the SLAS group. Statistical significance was found between groups (net difference: 1.16 kg/m^2^, 95% CI: 0.42–1.89, *p* = 0.001) (**Table 2**).

### Secondary outcomes

From baseline to treatment endpoint, a certain reduction was found in both waist circumference and hip circumference in either group. To be specific, the mean change in waist circumference in the LAS group was more significant than SLAS (−5.43 (SD: 5.08) versus −2.96 (SD: 5.34), *p* = 0.033), while the mean change in hip circumference showed no obvious difference between LAS and SLAS (−3.45 (SD: 4.12) versus −1.86 (SD: 3.94), *p* = 0.074). Furthermore, no significant difference was found in the change in WHR between the two groups. The detailed information regarding body measurements is presented in **Table 4**.

**Table 4:** Change of secondary outcomes after treatment.

	LAS (*n* = 42)	SLAS (*n* = 42)	Net difference and 95% CI	*p*-value
Body measurements				
Waist circumference (cm)	−5.43 (5.08)	−2.96 (5.34)	2.46 (0.20, 4.73)	0.033
Hip circumference (cm)	−3.45 (4.12)	−1.86 (3.94)	1.60 (0.16, 3.35)	0.074
WHR	−0.02 (0.03)	−0.01 (0.04)	0.01 (-0.03, 0.01)	0.23
Glucose and lipid metabolism				
TC (mmol/L)	0.06 (0.58)	0.20 (0.62)	0.15 (−0.41, 0.11)	0.27
TG (mmol/L)	−0.32 (1.43)	0.21 (0.53)	0.53 (0.06, 1.00)	0.027
LDL-C (mmol/L)	0.10 (0.72)	−0.02 (0.60)	0.01 (−0.21, 0.23)	0.95
HDL-C (mmol/L)	0.002 (0.19)	0.03 (0.22)	0.03 (−0.12, 0.06)	0.47

Data are in mean (SD) values.

HDL-C, low-density lipoprotein cholesterol; LAS, long-term acupoint stimulation; LDL-C, low-density lipoprotein cholesterol; SLAS, sham long-term acupoint stimulation; TC, total cholesterol; TG, triglyceride; WHR, waist-to-hip ratio.

At the end of treatment, no significant difference was observed in the change of lipid profiles (TC, HDL-C, or LDL-C) between the two groups, except for TG (**Table 4**). The mean reduction of TG in the LAS group was 0.32 (SD: 1.43) after the eighth treatment, while a slight elevation of TG was observed in the SLAS group (0.21 (SD: 0.53)); the difference was statistically significant (*p* = 0.027).

### Change of body adipose tissue

Based on voluntariness, the MRI evaluation for body adipose tissue was conducted on 53 participants (29 in the LAS group and 24 in the SLAS group, **Table 5**). VAT was assessed in the liver, pancreas, and kidney. After treatment, no significant difference was found between the two groups. SAT was investigated in four axial slices, namely, L2-L3, L3-L4, L4-L5, and L5-S1. At the endpoint of the eighth treatment, the SAT in L3-L4 was significantly reduced in the LAS group compared with the SLAS group (−49.74 (SD: 128.67) versus 13.30 (SD: 97.01), *p* = 0.043). Beyond that, no obvious difference was noticed in other segments between the two groups.

**Table 5:** Change in visceral adipose tissue and subcutaneous adipose tissue after the treatment.

	LAS (*n* = 29)	SLAS (*n* = 24)	*p*-value
Hepatic fat (mm^2^)	−0.01 (0.04)	−0.001 (0.02)	0.074
Pancreatic fat (mm^2^)	0.004 (0.04)	−0.003 (0.03)	0.50
Renal sinus fat (mm^2^)	−97.26 (164.03)	−161.11 (378.72)	0.29
SAT L2-L3 (cm^2^)	−38.50 (95.27)	3.25 (106.32)	0.14
SAT L3-L4 (cm^2^)	−49.74 (128.67)	13.30 (97.01)	0.043
SAT L4-L5 (cm^2^)	−52.23 (218.81)	−42.80 (211.52)	0.73
SAT L5-S1 (cm^2^)	−41.38 (121.64)	−14.08 (118.70)	0.34

Data are in mean (SD) values.

BMI, body mass index; LAS, long-term acupoint stimulation; SAT, subcutaneous adipose tissue; SLAS, sham long-term acupoint stimulation.

### Safety assessment

No abnormalities were reported in vital signs, laboratory tests, and 12-lead electrocardiograms throughout the whole trial. One participant in the LAS group complained of an uncomfortable and tingling sensation after the additional intervention. Relief was achieved after local hot compress. No other AEs were documented.

## Discussion

This clinical trial demonstrated that LAS could significantly improve body-weight control compared with sham control. In addition, the beneficial effect persisted for at least 3 months after the completion of the interventions. To the best of our knowledge, this is the first LAS clinical trial to use a PPDO suture as an embedding material. Both efficacy and safety profiles indicated that LAS with PPDO sutures could be used as a choice for body-weight management.

Compared with classical acupuncture, LAS is supposed to achieve a more significant effect by using fewer intervention times. The results of our trial confirm this statement. According to a previous systematic review, classical acupuncture could reduce additional body weight of 0.60 kg versus sham control (14). In our trial, by carrying out the procedure every 10 days, we achieved approximately three times the effect compared to the above data. In consideration of satisfactory safety, LAS may have the potential to replace classical acupuncture in body-weight management with support from further head-to-head trials.

The current guidelines recommend at least 5% weight loss as the treatment goal (7). At the end of treatment, one quarter of the participants have achieved the target. Based on the guidelines, this number is not satisfactory. However, in consideration of the relatively short intervention period (80 days), this result is still gratifying. Furthermore, our result in terms of body-weight change is substantially superior to a recently published randomized controlled trial in which the synthesized data involving 40 participants reported that body-weight reduction was 1.65 kg in the LAS group (32). Although our intervention period was 2 weeks longer, this may not be enough to explain the gap. The potential reason may be the distinct acupoint selection. Further studies could be considered to explore the specific mechanisms of chosen acupoints.

The mean body weight in the SLAS group was also slightly reduced compared with the baseline. This may result from the SLAS intervention. The comparator utilized in our trial was not a real placebo. The procedure still needed to insert the needle into the acupoints and acquire the needle sensation. Only the stimulation time was shortened. Thus, in order to obtain a comprehensive understanding of the effectiveness of LAS, a wait-list control group could be introduced in future trials.

The biocompatible materials of LAS are diverse. Catgut suture is one of the conventional choices. As one of the commonest materials, catgut embedding has been used in treating a variety of diseases other than obesity, such as insomnia, chronic urticaria, and postmenopausal osteoporosis (33–35). However, there were also certain AEs reported (36, 37). Therefore, we selected an innovative material, namely, PPDO suture, as our embedding material. Previous studies reported that PPDO sutures possessed satisfactory stability, biocompatibility, and biodegradability (26, 29). These features would guarantee sustaining the stimulation of acupoints while reducing the potential risk of AEs such as inflammation and discomfort. Our study did demonstrate this hypothesis. Included participants presented good tolerance to the PPDO suture, and no one rejected treatment due to AEs. This embedding material could be prioritized in future practice and extended to other indications of LAS.

Apart from traditional body-weight evaluation, we introduced MRI for objective evaluation of the change of adipose tissue. For visceral fat, the liver is the most affected organ by fat accumulation compared with the pancreas and kidney based on their responsibilities in lipid metabolism (38). This fact can also be supported by the TCM theory (39). The liver governs dispersion and regulates the metabolism of body fluids. Impairment of function would cause accumulation of excess water and grain in the liver, which corresponds to fat accumulation in modern medicine. Based on our results, LAS showed no obvious effect on visceral fat but could significantly reduce subcutaneous fat around the waist (L3-L4) after eight times of treatment. This finding revealed that the relatively short-term improvement in body weight by LAS may come from the decrease in SAT. Furthermore, according to our results of lipid profiles, LAS significantly reduced the level of TG but showed no obvious effect on LDL-C and TC. Considering that TG is one of the main components stored in adipose tissue, we speculated that the short-term beneficial effect of LAS on body weight may result from its regulation of TG metabolism rather than TC or LDL-C. Previous acupuncture studies utilized different acupoint regimes and showed positive effects on TC and LDL-C (40). Hence, we may consider modification of our acupoint regime in future studies, hoping to find a new acupoint combination that benefits cholesterol metabolism. Based on our findings, abdominal fat accumulation is a characteristic of central obesity and serves as an independent risk factor for cardiovascular outcomes (41, 42). In further trials, it will be worth assessing the effectiveness of LAS in this population.

The mechanisms underlying LAS for body-weight control still lack relevant studies. Considering that prolonged needle sensation is the main characteristic of LAS, we speculated that the potential mechanisms are similar. In general, acupuncture treatment involves four main routes, namely, lipid and energy metabolism, inflammatory response, appetite regulation, and adipose-tissue transformation (43). For instance, electroacupuncture on CV12 and Guanyuan (CV4) can regulate the ratio of adiponectin and leptin in obese Zucker diabetic fatty rats and then further restore the balance of glucose and lipid metabolism (44). Huang et al. found that electroacupuncture on CV12, ST40, Zusanli (ST36), and CV4 could activate the silent information regulation factor 1/nuclear factor-kappa B signaling pathway in obese rats and reduce the expression of downstream inflammatory factors (45). Ghrelin and leptin are counter hormones in regulating appetite (46). A previous animal study found that acupuncture could regulate a diet’s rhythm by reducing ghrelin levels (47). A randomized controlled trial also reported that acupuncture’s effect on weight control may result from its regulation of the balance between leptin and ghrelin (48). Other studies also reported that acupuncture could promote white adipose tissue browning *via* enhancing the expression of uncoupling protein-1 (47, 49). To verify these mechanisms and explore the potential amplifying effect brought by LAS, basic studies utilizing real LAS therapy should be supplemented.

Our study provided a general acupoint regime of LAS therapy for weight control. However, factors such as demographic characteristics, acupuncture techniques, and causes of overweight/obesity may interfere with efficacy (50, 51). In clinical practice, doctors are encouraged to adjust the acupoint regime flexibly based on the patients’ status. Further studies can also be considered in exploring individualized acupoint selection in treating overweight/obesity.

Our study has certain limitations. Firstly, participant recruitment was not only limited to simple obesity. Nearly half of the participants possessed BMI levels between 24 and 28 kg/m^2^. This population may weaken the overall efficacy of LAS treatment. Secondly, only body measurements and lipid metabolism were evaluated in this study. Obesity-related hormones, such as leptin and ghrelin, were not involved. Hence, we could not explain the potential mechanisms of LAS. The sample size of this study was limited. The observed effect size was lower than expected. According to the study results, a larger-scale clinical trial is needed with at least 92 participants in each group. Furthermore, our clinical trial was a simple sham-controlled study. No active intervention or blank control was introduced. Thus, we could not estimate the specific position of LAS in the management algorithm of body weight. Further head-to-head studies are suggested. Lastly, the evaluation for lifestyle modification only served as a compliance indicator in this trial. The lack of lifestyle data partially limited the explanation of study results. Real-time monitoring and documenting of participants’ daily lifestyle data *via* smart wearable devices would be a considerable solution.

In summary, the modified acupuncture technique, specifically, LAS with PPDO suture, was superior to sham control in controlling body weight after 80 days of treatment. Also, the therapeutic effect persisted throughout the 3-month follow-up period. The benefit may result from the reduction of SAT. Further studies could focus on mechanism exploration and head-to-head comparison with standard medications.

## Data availability statement

The original contributions presented in the study are included in the article/**Supplementary Material**. Further inquiries can be directed to the corresponding author.

## Ethics statement

The studies involving human participants were reviewed and approved by Clinical Trial Institutional Review Board of Longhua Hospital Affiliated to Shanghai University of Traditional Chinese Medicine. The patients/participants provided their written informed consent to participate in this study.

## Author contributions

All listed authors met the requirements for authorship. LD and MW designed the trial, interpreted the data, and drafted the manuscript, and were considered as co-first authors. K-PZ, LW, and H-MZ recruited the participants and performed treatments. C-BL conducted an MRI assessment and analysis. W-JZ and S-GZ coordinated the conduct of the study. GJ initiated the trial and supervised the execution. All authors read and approved the final manuscript.

## Funding

This work was supported by the Shanghai Three-year Action Plan for Accelerating the Development of Traditional Chinese Medicine (No. ZY (2018-2020)-CCCX-2002-01) and the Clinical Research Plan of SHDC (No. SHDC12017X16). The funders had no role in the study design, data collection and analysis, the decision to publish, or the preparation of the manuscript.

## Acknowledgments

We thank the research assistants and nurses from the three medical centers for guaranteeing the smooth proceeding of this clinical trial. We also thank Dr. Ying Shan from the Clinical Research Academy, Peking University Shenzhen Hospital, for statistical analysis support.

## Conflict of interest

The authors declare that the research was conducted in the absence of any commercial or financial relationships that could be construed as a potential conflict of interest.

## Publisher’s note

All claims expressed in this article are solely those of the authors and do not necessarily represent those of their affiliated organizations, or those of the publisher, the editors and the reviewers. Any product that may be evaluated in this article, or claim that may be made by its manufacturer, is not guaranteed or endorsed by the publisher.
