# Supplementary material for: Modified acupuncture therapy, long-term acupoint stimulation versus sham control for weight control: a multicenter, randomized controlled trial
Source: Front Endocrinol (Lausanne). 2022 Jul 28;13:952373. doi: 10.3389/fendo.2022.952373 (PMC9365970; doi:10.3389/fendo.2022.952373)
Supplement: Supplementary file 1 [file DataSheet_1.pdf]

Supplementary Material

1 Supplementary File

1.1 Assessment method of change of body adipose tissue by using magnetic resonance imaging

In this study, we performed a 45-minute 3-Tesla magnetic resonance imaging (MRI) (Ingeina 3.0 T, Siemens) to evaluate the visceral adipose tissue (VAT) and subcutaneous adipose tissue (SAT) at baseline and treatment endpoint. The scanner utilized a 3D modified DIXON (mDIXON) imaging without gaps (2mm thickness and 2mm of spacing), fast-low-angle shot (FLASH) sequence with a multi-echo two-excitation pulse sequence for phase-sensitive encoding of fat and water signals (TR, 3.6ms; TE1, 1.19ms; TE2, 2.3ms; FOV 520*440*80mm; 2*1.4*1mm voxel size). Four images of the phantoms were generated, including in-phase, out-phase, fat and water phase. A breath-hold technique was used to avoid motion artifacts when the chest and abdomen were scanned. We quantified SAT and VAT using the 3DSlicer software. Subcutaneous fat depots: We calculated mean SAT area from axial slices: L5-S1, L4-L5, L3-L4 and L2-L3. We drew a continuous line over the deep-SAT and superficial-SAT, and calculated mean SAT area. Hepatic fat content: We quantified the percentage of hepatic fat using ImageJ software. We calculated mean percentage from four 2D slices (3cm intervals divided into quarters) by utilizing the region of interest (ROI) approach, which is based on measurements of tissue densities (fat/fat + water) using the Fat ratio calculation. We divided each slice into quarters, and chose ROIs in each of the four quarters in order to represent the entire liver. We determined the mean percentage of fat for each slice and quarter, and then calculated the mean percentage of fat in the liver as a whole. Pancreatic Fat: We found the largest cross section of the pancreas, draw the maximum area with 3DSlicer and calculate the Pancreatic fat percentage. Renal sinus fat: We found the largest cross section of the renal sinus, draw the maximum area with 3DSlicer and calculate the renal sinus fat percentage.

2 Supplementary Figures and Tables

2.1 Supplementary Figures

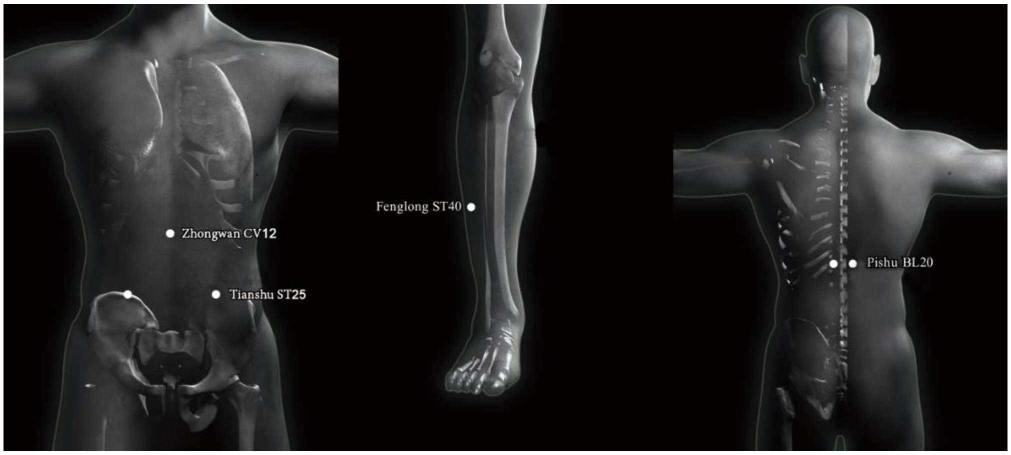

Supplementary Figure 1. The specific anatomical locations of selected acupoints.

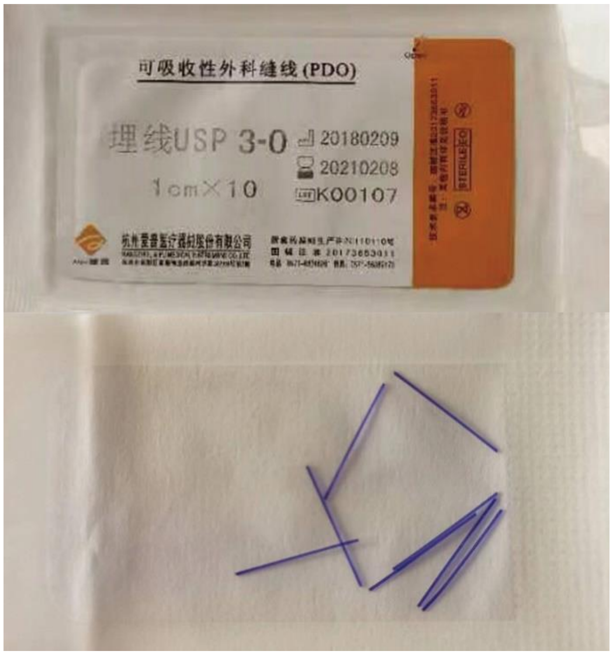

Supplementary Figure 2. The size of implanted PPDO suture.

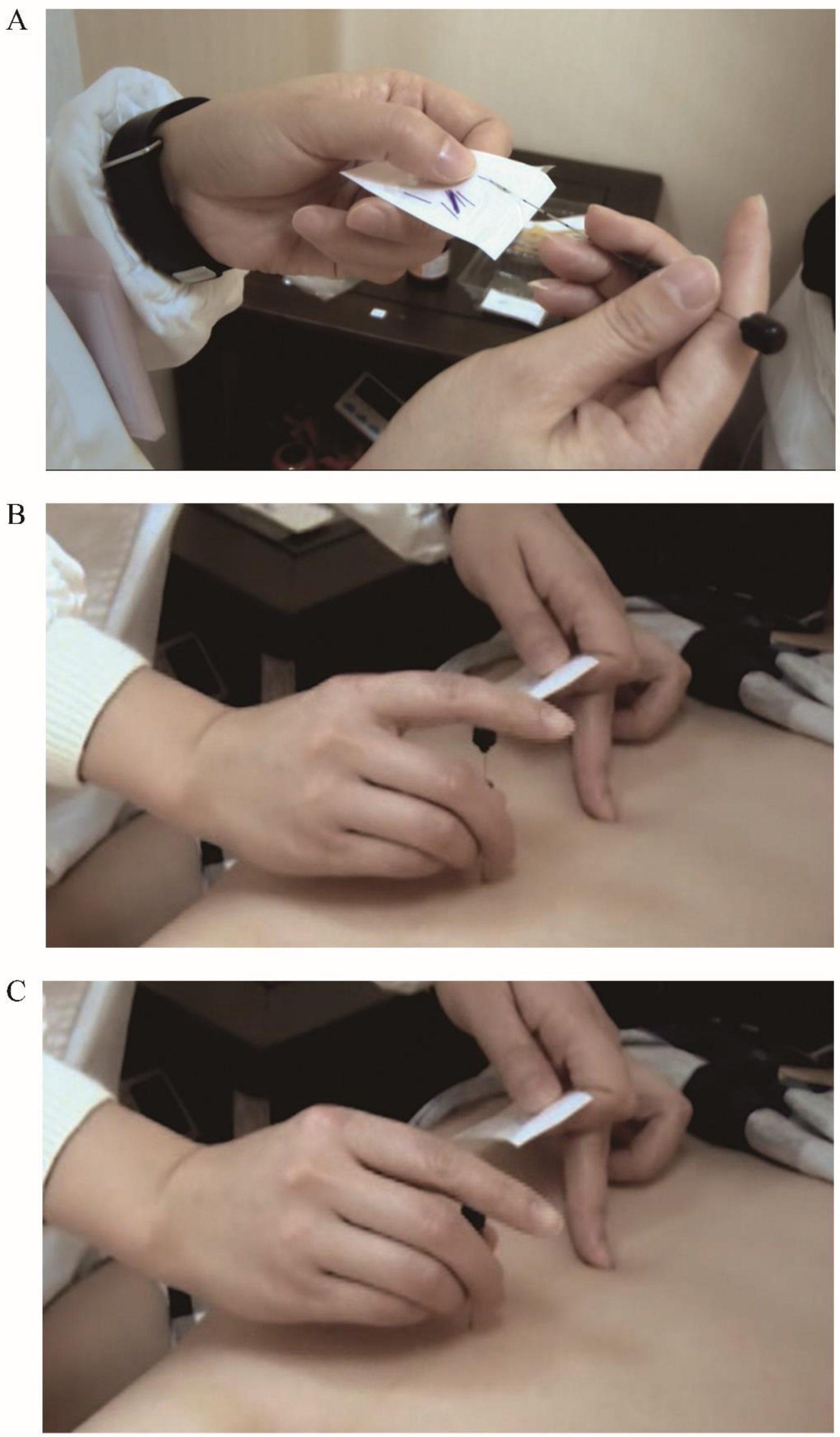

Supplementary Figure 3. The key procedures of LAS therapy. (A) Amicrobic PPDO suture (size: 3-0) was firstly placed in the tip of a disposable embedding needle, before the stylet, (B) The needle was inserted at a depth of 1.5-2.0 cm into the acupoint, (C) Acupuncturist would gradually pull out the tubing and push in the stylet at the same time.

2.2 Supplementary Tables

Supplementary Table 1. CONSORT 2010 checklist of information to include when reporting a randomized trial

Section/Topic	Item No	Checklist item	Reported on page No
**Title and abstract**			
	1a	Identification as a randomised trial in the title	1
	1b	Structured summary of trial design, methods, results, and conclusions (for specific guidance see CONSORT for abstracts)	1-2
**Introduction**			
Background and objectives	2a	Scientific background and explanation of rationale	2
	2b	Specific objectives or hypotheses	2
**Methods**			
Trial design	3a	Description of trial design (such as parallel, factorial) including allocation ratio	2-3
	3b	Important changes to methods after trial commencement (such as eligibility criteria), with reasons	Not applicable
Participants	4a	Eligibility criteria for participants	3
	4b	Settings and locations where the data were collected	2
Interventions	5	The interventions for each group with sufficient details to allow replication, including how and when they were actually administered	3-4
Outcomes	6a	Completely defined pre-specified primary and secondary outcome measures, including how and when they were assessed	4
	6b	Any changes to trial outcomes after the trial commenced, with reasons	Not applicable
Sample size	7a	How sample size was determined	4

	7b	When applicable, explanation of any interim analyses and stopping guidelines	Not applicable
Randomisation:			
Sequence generation	8a	Method used to generate the random allocation sequence	4
	8b	Type of randomisation; details of any restriction (such as blocking and block size)	4
Allocation concealment mechanism	9	Mechanism used to implement the random allocation sequence (such as sequentially numbered containers), describing any steps taken to conceal the sequence until interventions were assigned	4
Implementation	10	Who generated the random allocation sequence, who enrolled participants, and who assigned participants to interventions	4
Blinding	11a	If done, who was blinded after assignment to interventions (for example, participants, care providers, those assessing outcomes) and how	4
	11b	If relevant, description of the similarity of interventions	3-4
Statistical methods	12a	Statistical methods used to compare groups for primary and secondary outcomes	4-5
	12b	Methods for additional analyses, such as subgroup analyses and adjusted analyses	Not applicable
**Results**			
Participant flow (a diagram is strongly recommended)	13a	For each group, the numbers of participants who were randomly assigned, received intended treatment, and were analysed for the primary outcome	5, Figure. 1
	13b	For each group, losses and exclusions after randomisation, together with reasons	5, Figure. 1
Recruitment	14a	Dates defining the periods of recruitment and follow-up	5
	14b	Why the trial ended or was stopped	Not applicable
Baseline data	15	A table showing baseline demographic and clinical characteristics for each group	Table 2

Numbers analysed	16	For each group, number of participants (denominator) included in each analysis and whether the analysis was by original assigned groups	Table 3-5
Outcomes and estimation	17a	For each primary and secondary outcome, results for each group, and the estimated effect size and its precision (such as 95% confidence interval)	5-7, Table 3-5
	17b	For binary outcomes, presentation of both absolute and relative effect sizes is recommended	Not applicable
Ancillary analyses	18	Results of any other analyses performed, including subgroup analyses and adjusted analyses, distinguishing pre-specified from exploratory	Not applicable
Harms	19	All important harms or unintended effects in each group (for specific guidance see CONSORT for harms)	7
**Discussion**			
Limitations	20	Trial limitations, addressing sources of potential bias, imprecision, and, if relevant, multiplicity of analyses	9
Generalisability	21	Generalisability (external validity, applicability) of the trial findings	8-9
Interpretation	22	Interpretation consistent with results, balancing benefits and harms, and considering other relevant evidence	8-9
**Other information**			
Registration	23	Registration number and name of trial registry	2
Protocol	24	Where the full trial protocol can be accessed, if available	2
Funding	25	Sources of funding and other support (such as supply of drugs), role of funders	9

Supplementary Table 2. STRICTA 2010 checklist of information to include when reporting interventions in a clinical trial of acupuncture

**Item**	**Detail**	**Reported on page No.**
1. Acupuncture rationale	1a) Style of acupuncture (e.g. Traditional Chinese Medicine, Japanese, Korean, Western medical, Five Element, ear acupuncture, etc)	2
	1b) Reasoning for treatment provided, based on historical context, literature sources, and/or consensus methods, with references where appropriate	2-3
	1c) Extent to which treatment was varied	2-3
2. Details of needling	2a) Number of needle insertions per subject per session (mean and range where relevant)	3
	2b) Names (or location if no standard name) of points used (uni/bilateral)	3, Table 1, Figure S1
	2c) Depth of insertion, based on a specified unit of measurement, or on a particular tissue level	3
	2d) Response sought (e.g. *de qi* or muscle twitch response)	3
	2e) Needle stimulation (e.g. manual, electrical)	3
	2f) Needle retention time	3
	2g) Needle type (diameter, length, and manufacturer or material)	3
3. Treatment regimen	3a) Number of treatment sessions	3-4
	3b) Frequency and duration of treatment sessions	3-4
4. Other components of treatment	4a) Details of other interventions administered to the acupuncture group (e.g. moxibustion, cupping, herbs, exercises, lifestyle advice)	4
	4b) Setting and context of treatment, including instructions to practitioners, and information and explanations to patients	4
5. Practitioner background	5) Description of participating acupuncturists (qualification or professional affiliation, years in acupuncture practice, other relevant experience)	3

6. Control or comparator interventions	6a) Rationale for the control or comparator in the context of the research question, with sources that justify this choice	3
	6b) Precise description of the control or comparator. If sham acupuncture or any other type of acupuncture-like control is used, provide details as for Items 1 to 3 above.	3
